# Perspectives on the *Pseudomonas aeruginosa* Type III Secretion System Effector ExoU and Its Subversion of the Host Innate Immune Response to Infection

**DOI:** 10.3390/toxins13120880

**Published:** 2021-12-09

**Authors:** Kierra S. Hardy, Maxx H. Tessmer, Dara W. Frank, Jonathon P. Audia

**Affiliations:** 1 Department of Microbiology and Immunology, University of South Alabama College of Medicine, Mobile, AL 36608, USA; ksh1004@jagmail.southalabama.edu; 2 Center for Lung Biology, University of South Alabama College of Medicine, Mobile, AL 36608, USA; 3 Department of Systems Biology, Harvard Medical School, Boston, MA 02115, USA; 4 Department of Chemistry, University of Washington, Seattle, WA 98195, USA; mhtessmer@gmail.com; 5 Department of Microbiology and Immunology, Medical College of Wisconsin, Milwaukee, WI 53226, USA

**Keywords:** *Pseudomonas aeruginosa*, ExoU, phospholipase A_2_, pneumonia, innate immunity, amyloids, inflammasomes

## Abstract

*Pseudomonas aeruginosa* is an opportunistic, Gram-negative pathogen and an important cause of hospital acquired infections, especially in immunocompromised patients. Highly virulent *P. aeruginosa* strains use a type III secretion system (T3SS) to inject exoenzyme effectors directly into the cytoplasm of a target host cell. *P. aeruginosa* strains that express the T3SS effector, ExoU, associate with adverse outcomes in critically ill patients with pneumonia, owing to the ability of ExoU to rapidly damage host cell membranes and subvert the innate immune response to infection. Herein, we review the structure, function, regulation, and virulence characteristics of the T3SS effector ExoU, a highly cytotoxic phospholipase A_2_ enzyme.

## 1. Introduction

*Pseudomonas aeruginosa* is a Gram-negative, rod-shaped bacterium found in soil and water. Similar to other pseudomonads and mucosal pathogens, *P. aeruginosa* possesses a single polar flagellum for motility [1]. Due to its biosynthetic capacity, *P. aeruginosa* thrives in environments with minimal nutrients and under diverse physical conditions, allowing persistence in hospital and community settings [2]. As an important cause of nosocomial pneumonia globally, *P. aeruginosa* is classified as an ESKAPE (*Enterococcus faecium, Staphylococcus aureus, Klebsiella pneumoniae, Acinetobacter baumannii, Pseudomonas aeruginosa,* and *Enterobacter* species) pathogen listed as critical priority for new research and drug development by the World Health Organization [3,4,5]. Healthcare infections associated with *P. aeruginosa* exceed 51,000 cases annually in the United States and are responsible for 40% of deaths of patients with ventilator-associated pneumonia (VAP) [6,7,8,9,10]. *P. aeruginosa* causes both acute and chronic infections. Severe burn, mechanically ventilated, and immunocompromised patients are particularly susceptible to acute *P. aeruginosa* infections [11,12]. Patients with pre-existing lung disease, such as cystic fibrosis, are susceptible to chronic *P. aeruginosa* infections [11,12].

**Figure 1:**
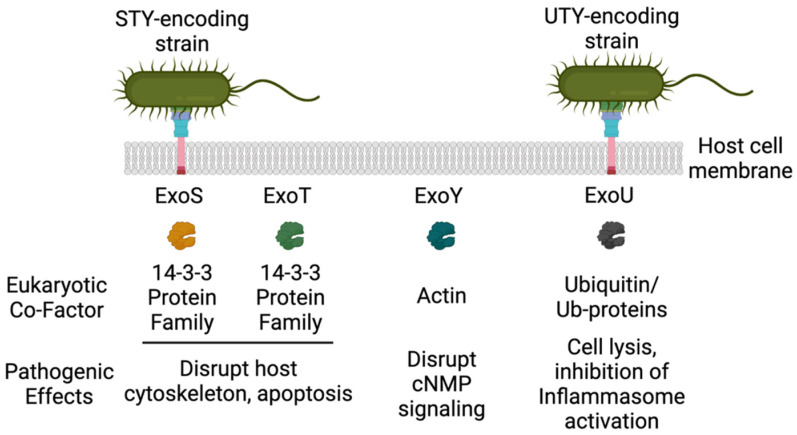
The *P. aeruginosa* T3SS effectors, their activators, and host cell effects (Created with BioRender.com).

*P. aeruginosa* is a pathogen commonly isolated from patients with acute respiratory infections, which result from airway epithelial damage due to mechanical ventilation [13,14]. Despite improvements in mechanical ventilation strategies, such as reduced tidal volumes, patients often suffer injury to their innate lung barriers due to the endotracheal tubing, allowing the pathogen to attach and colonize mucous membranes [13,15]. Epithelial and alveolar macrophages become activated and damaged upon *P. aeruginosa* colonization of the airways [16,17]. Neutrophils and phagocytic-mediated clearance mechanisms are also impaired by a diverse array of pathogen-encoded virulence factors [18,19]. *P. aeruginosa* uses a wide array of virulence mechanisms to cause disease in humans (for excellent reviews see [20,21]). The *P. aeruginosa* type III secretion system (T3SS) is one of the most notorious of these virulence mechanisms, and aids in colonization of the airway and in immune avoidance [22]. To date, there are at least four well-characterized effector proteins referred to as Exoenzymes: ExoU, S, T, and Y [13]. Once delivered into the host cell cytoplasm, these effector proteins become activated through interactions with their cognate eukaryotic cofactors (Figure 1). Interestingly, *P. aeruginosa* clinical and environment strains vary in the cadre of T3 effectors encoded, which defines the pathophysiology of a given strain/isolate [8,23,24]. The relative distribution of T3 effectors is: ~35% ExoU, ~65% ExoS, ~100% ExoT, and ~90% ExoY [25]. ExoS and ExoT were the first *P. aeruginosa* T3 effectors identified and are bifunctional enzymes possessing GTPase activating protein (GAP) and ADP-ribosyltransferase activities that trigger pathological rearrangements in the host cell cytoskeleton [26]. These activities lead to cytotoxicity (ExoS) or host cell cytoskeletal rearrangements that limit phagocytosis (ExoT). Intoxication of cells by ExoS is characterized by cell death that takes several hours to manifest and results ultimately in apoptosis [27]. ExoY is a promiscuous nucleotidylyl cyclase [28,29] that disrupts inter-endothelial gap junctions and promotes hyperphosphorylation and release of tau and amyloid-β [30,31,32,33]. ExoU was identified based on the observation that several clinical isolates possessed a rapid or acute cytotoxic response when co-cultivated with epithelial cells, which was distinct from the pattern of cell death induced by ExoS [34]. *P. aeruginosa* ExoU has been associated with severe disease outcomes in humans and in animal infection models [10,35]. The goal of this review is to provide an update on the role of ExoU in pathogenesis in the context of structure, function, and virulence relationships.

## 2. ExoU

Of the four effector proteins, ExoU is the most acutely cytotoxic. ExoU was identified in 1997 as an effector secreted by *P. aeruginosa* strain PA103 [34,36]. The gene encoding ExoU was identified as part of a mutant library screen and verified by peptide sequence analyses. ExoU is a 74-kDa (686 amino acid) protein that has a 5-residue amino-terminal sequence similar to ExoS and ExoT, which may be important for targeting to the T3SS [34]. Unlike the other effectors, *exoU* and its chaperone, *spcU*, are thought to have been acquired through horizontal gene transfer and reside on an 81-kb pathogenicity island [37]. In support of this hypothesis, yeast recombinational cloning and sequence analysis revealed *exoU* has a lower G + C content (59%) compared to *exoS* encoded by strain PAO1 (67%) and flanking elements homologous to IS407 [37]. In epithelial cells, ExoU expression is associated with rapid cellular lysis that occurs within 3 h of co-culture with *P. aeruginosa*. Cytotoxicity was so rapid that to mechanistically understand how ExoU functioned intracellularly, a system using *Saccharomyces cerevisiae* as a surrogate expression host was developed. ExoU synthesis could be controlled in yeast experimental model and the yeast cell wall protected the cell long enough to facilitate visualization of ExoU-induced damage to intracellular membranes and vacuolar fragmentation [38]. The addition of phospholipase inhibitors such as methyl arachidonyl fluorophosphonate (MAFP) significantly decreased ExoU cytotoxicity in yeast and mammalian cells. Combined with the nucleotide alignment analyses revealing a PLA domain similar to patatin, cPLA_2_, and iPLA_2_ [38], ExoU was postulated to function as a phospholipase hydrolyzing neutral lipids and phospholipids [38,39,40]. This model reconciled the rapid cytotoxicity in vitro and significant tissue destruction mediated by ExoU producing strains in animal models of infection.

### 2.1. Structure-Activity Relationships of ExoU

ExoU is a relativity large protein (686 amino acids, 74 kDa) making domain mapping somewhat complex. It was known that the protein likely possessed multiple functional domains even before crystal structures were available. Early studies of truncated proteins transfected into Chinese Hamster Ovary cells suggested that N- and C-terminal regions were necessary but not sufficient for toxicity [41]. Sato et al. and Philips et al. independently identified and verified, by site-specific mutagenesis studies, that ExoU was, indeed, part of the patatin family of phospholipases [38,39]. Patatin is a major storage protein in potatoes and other tubers that hydrolyzes phospholipids, *p*-nitro-phenyl esters, and monoacylglycerols [40,42]. Environmental stress or pathogenic infection results in patatin enzyme activation [40,42]. The patatin family of enzymes share functional similarity to eukaryotic cPLA_2_ and iPLA_2_, and possess PLA_2_ activity that hydrolyzes the acyl group at the *sn-2* position of phospholipids, resulting in release of free fatty acids, such as lysophospholipids and arachidonic acid. There are three major conserved regions between ExoU, patatin, cPLA_2_, and iPLA_2_: a glycine-rich motif (oxyanion hole, G-G-X-R/K), a hydroxylase motif with a catalytic serine (G-X-S-X-G) and a catalytic aspartate motif (D-X-G/A). The latter two are commonly referred to as a serine-aspartate catalytic dyad, S142, and D344 for ExoU [38,43,44]. The catalytic aspartate is postulated to remove a proton from the catalytic serine, which allows the serine nucleophile to attack the carbonyl group of the ester located in the *sn*-2 position on the *sn*-glycerol-3-phosphate backbone of the phospholipid. The glycine-rich motif stabilizes the transition state. This stabilized state precedes collapse of the tetrahedral intermediate and release of lysophospholipid, leaving a serine-acyl intermediate. A similar mechanism is thought to apply to the hydrolysis of the serine-acyl intermediate. In this case, the catalytic aspartate activates water for a nucleophilic attack of the carbonyl carbon of the ester. Alanine substitutions at S142 or D334 render ExoU non-toxic and unable to cleave phospholipids. These and earlier studies identifying the binding domain for the ExoU chaperone, SpcU [45], indicated that the amino-terminal half of ExoU was required for T3SS secretion and contained the catalytic domain.

Functional mapping studies have identified several important regions within the ExoU C-terminus and downstream of the catalytic domain (positions past D344). Rabin et al. utilized peptide fusions to green fluorescent protein to map a plasma membrane localization domain (MLD) that resides between residues 550–687 [46]. Analysis of linker-insertions suggested that C-terminal residues were somehow associated with recognition of the eukaryotic co-factor protein required for activity [47]. Error-prone mutagenesis studies identified specific amino acid residues I609, Q623, N627, I654, R661, and A678 that appeared to play roles in both membrane localization and catalysis. Additionally, these studies indicated that a non-proteinaceous co-factor may contribute to ExoU phospholipase activity [48]. Stirling et al. demonstrated that a small C-terminal region (679–683) controls both membrane localization and the addition of diubiquitin at lysine 178 [49]. Covalent modification of ExoU was not required for toxicity, however. Overall, the C-terminal region of ExoU is important for both activity and the co-factor interactions that are postulated to facilitate conformational changes from an apoenzyme (inactive) to a holoenzyme (active) state [50].

### 2.2. Crystal Structure of ExoU

**Figure 2:**
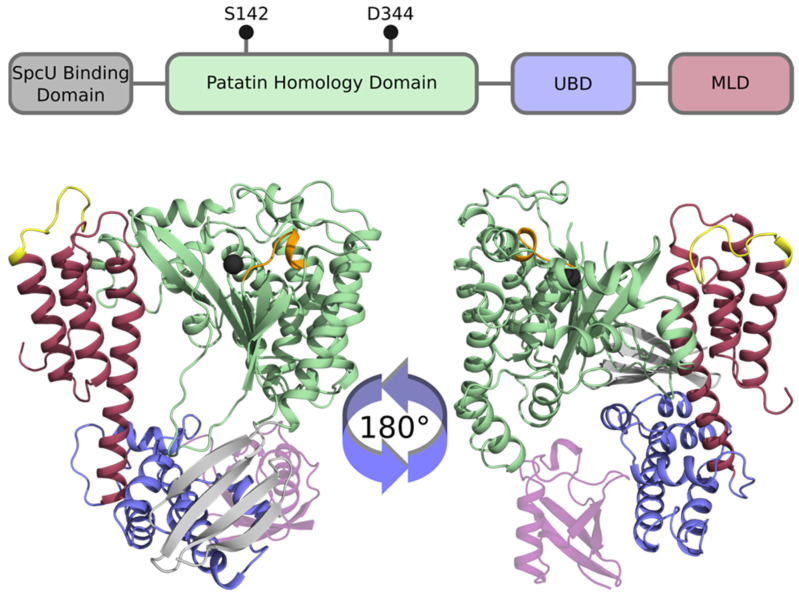
The domain structure of ExoU. (**Top**) Cartoon diagram of the domain structure of ExoU. The N-terminal SpcU binding domain is shown in gray, followed by the catalytic patatin homology domain. Black pins indicate the approximate location of the catalytic dyad, S142 and D344. The ubiquitin-binding domain (UBD, bridging domain) is shown in blue followed by the membrane localization domain (MLD) in red. (**Bottom**) ExoU structural model. Functional domains are highlighted using the same colors as A with the addition of ubiquitin (transparent purple). The catalytic serine is marked as a black sphere and the catalytic aspartate is not modeled. Adjacent to the sphere is the conserved G-G-X-R/K motif shown in orange. The membrane interacting loop 3 of the C-terminal 4-helix bundle is highlighted in yellow. Loops missing from the crystal structures were modeled as previously described [54].

The functional mapping data and domain structure of ExoU was resolved when two independent groups published crystal structures of ExoU in complex with its chaperone, SpcU [51,52]. The structures are in good agreement with each other and show a complex multidomain organization. Figure 2 shows a structural diagram and ribbon models of ExoU based on the published structural solution data discussed herein. Domain 1 consists of a SpcU chaperone-binding motif located at the N-terminus comprising residues 55–101 [52]. Binding of SpcU to ExoU keeps the protein in an inactive state in the bacterial cytosol [52]. In addition, SpcU guides ExoU to the T3SS where it unfolds and is secreted through the injectisome into the host cell cytosol [53]. Following the chaperone-binding domain is the patatin-like domain which encodes the PLA_2_, residues 106–471. Within this region is the serine-aspartate catalytic dyad that is required for ExoU cytotoxicity [52]. At the C-terminus is a bridging domain, residues 480–580, and a membrane localization domain (MLD) comprising domains 3 and 4, residues 588–687 [51,52]. The MLD is a 4-helix bundle connected by several loop regions. Approximately 25% of the amino acid residues cannot be localized from the electron density map, including the catalytic aspartate at position 344, suggesting a fair amount of structural flexibility.

### 2.3. ExoU PLA_2_ Activity and Regulation by Eukaryotic Co-factors

Eukaryotic cell models and in vitro biochemistry techniques have been useful tools to characterize the regulation of ExoU PLA_2_ activation. Cloning and expression of ExoU as a recombinant protein revealed that the predicted PLA_2_ activity was readily detectable in vivo and in cell culture models. However, in vitro assays showed that the predicted PLA_2_ activity was only detectable when extracts of a eukaryotic cell (e.g., mammalian cells or yeast) were added to reactions containing phospholipid substrates. These experiments suggested that a eukaryotic factor was needed for ExoU activation, a regulatory feature that has also been described for ExoS, ExoT, and ExoY. To identify the eukaryotic activator of ExoU, Sato et al. performed biochemical enrichment experiments using cell extracts and selecting fractions that activated ExoU. These studies led to the discovery that SOD1 from either bovine or yeast sources activated ExoU in a dose-dependent manner [55]. However, SOD1 was not saturable in kinetic assays, suggesting that only part of the enriched fraction could activate ExoU [56,57]. Further analyses demonstrated that activation of ExoU by SOD1 was due a small subpopulation of molecules that was modified or contaminated with ubiquitin. Anderson et al. demonstrated that ubiquitin itself is a potent activator of ExoU PLA_2_ activity. Polyubiquitin and ubiquitinylated proteins also activate ExoU [57]. Ubiquitin is an abundant protein in all eukaryotic cells (up to 5% of total protein) but is absent in prokaryotes [58], making it an ideal activator of a protein with lethal activity. With crystal structures in hand, Tessmer et al. used computational, biophysical and biochemical approaches to map the ubiquitin interface to alpha helix 18 of ExoU and the hydrophobic face of ubiquitin [59,60]. Key residues of ExoU include T519, T522, V523, and S527 within the bridging domain [61].

Phosphatidylinositol 4,5-bisphosphate [PI(4,5)P_2_] has also been identified as both an activator and substrate for ExoU [51,62,63,64]. PI(4,5)P_2_ is also only present in eukaryotes and makes up approximately 1% of the total phospholipid content [65]. PI(4,5)P_2_ is associated with the inner leaflet of the cellular plasma membrane and participates in a variety of membrane-associated functions including motility, trafficking, signaling, and phagocytosis [65]. ExoU binds to liposomes containing 0.5% PI(4,5)P_2_ with an affinity of approximately 182 µM whereas liposomes lacking PI(4,5)P_2_ displayed a dissociation constant of 1.1 mM [66]. A PI(4,5)P_2_ binding motif is located within loop 3 of the C-terminal 4 helix bundle. Residue R661 serves as a key positively charged amino acid that interacts the negatively charged phosphate moieties of PI(4,5)P_2_ [62]. Using site-directed spin-labeling electron paramagnetic resonance spectroscopy, sites consisting of a basic-hydrophobic motif (residues 660–670, ARGFLRFGKPL) were found to be motionally dynamic when ExoU was analyzed with buffer alone (apo state), but transitioned to a more restricted state when ExoU was incubated with liposomes and diubiquitin (holo state, [60]). Accessibility to NiEDDA relaxation reagents further suggest that the loop inserts into the phospholipid bilayer, effectively anchoring ExoU to substrate [60]. PI(4,5)P_2_ may also serve to oligomerize ExoU to mediate optimal enzymatic activity [64]. In terms of regulating biochemical function, high affinity binding of ExoU to PI(4,5)P_2_ is important for plasma membrane targeting and maximal enzyme activity. Biologically, this interaction is likely linked to cleavage, disassembly of membrane focal adhesion complexes and cytoskeletal collapse associated with the early stages of ExoU intoxication [67].

Structural biology and biochemical studies of ExoU activation serve as a scaffold for the rational design of inhibitors aimed at reducing ExoU-mediated cytotoxicity in cells. Strategies to develop novel therapeutics are critical to combating infections with *P. aeruginosa*; a multi-drug resistant ESKAPE pathogen. Lee and colleagues utilized a high-throughput cell-based assay to screen chemical compounds and identified an ExoU PLA_2_ activity specific inhibitor named Pseudolipasin A [68]. Pseudolipasin A (PsA) did not affect bacterial growth or ExoU secretion through the T3SS, but it specifically targeted the enzymatic activity of ExoU, reducing the virulence of *P. aeruginosa* strains encoding ExoU. Treatment with PsA provided yeast, amoeba, and mammalian cells protection from ExoU-mediated killing [68]. It will be important for future studies to test PsA in pre-clinical animal infection models to determine any potential therapeutic utility in vivo. In 2014, a group of researchers identified a small series of arylsulfonamide compounds that inhibit ExoU cytotoxicity in a yeast-based screening assay [69]. *S. cerevisiae* was transformed with an ExoU expressing plasmid regulated by a copper-inducible expression system (pDH105). Cell viability measured 48 h post treatment with 5 µM of the different compounds identified arylsulfonamide [69]. Coupling amines with sulfonyl chlorides produced several compounds that inhibited ExoU-mediated cytotoxicity in yeast and mammalian cells with inhibitory activity similar to PsA [69]. Whether these arylsulfonamide compounds inhibit ExoU PLA_2_ activity, cofactor binding, or alter ExoU conformational state remains to be determined [70]. Furthermore, two potential ExoU inhibitors have been identified, PsA and arylsulfonamide; in-depth analyses of structure-activity relationships are pertinent to further characterize these inhibitors or to generate new inhibitors targeting ExoU.

### 2.4. ExoU Orthologues

Ubiquitin-mediated activation of patatin-like enzymes was postulated to be a conserved process in bacteria. Anderson et al. performed an in silico bioinformatics survey of bacterial genomic sequences to identify patatin-like enzymes [66]. The number of candidates were narrowed with additional search criteria that included possession of a T3 or T4SS for cytoplasmic delivery of the enzyme and a size exceeding 500 residues. Enzymes from bacteria with different lifestyles were purified, tested biochemically, or analyzed in prokaryotic and eukaryotic toxicity assays. Common properties included the requirement of ubiquitin to detect enzymatic activity in vitro, toxicity for bacterial cells when ubiquitin was provided by a compatible, inducible expression construct, and the ability to cause membrane damage or to be expressed in eukaryotic host cells. Activity enhancement by or binding to liposomes containing PI(4,5)P_2_ was a variable property suggesting that subfamilies of ExoU-orthologs exist. When enzymes encoded by potential pathogens (*Achromobacter*, *Aeromonas*, *Legionella*, *Rickettsia*, or *Vibrio* genera) were biochemically and biologically queried, several were classified as ubiquitin-activated, including enzymes from *Achromobacter xylosoxidans* and *Aeromonas diversa*. Based on modeling studies, the ExoU orthologue from *A. diversa* is structurally and functionally similar to ExoU. In contrast, the enzyme encoded by *A. xylosoxidans,* AxoU, maintains structural similarity only with the ExoU patatin domain. *A. xylosoxidans* is an emerging pathogen in cystic fibrosis patients and an opportunistic pathogen in immunocompromised individuals. Pathogenesis of this organism has been associated with a T3SS but the role of AxoU or other effectors is unclear [71].

### 2.5. ExoU PLA Activity and Pathogenesis

Besides several *Pseudomonas* species, other bacterial genera utilize PLA activity for pathogenesis. *Rickettsia prowazekii* protein, RP534, is an ExoU orthologue that possesses PLA_1_, PLA_2_, and Lyso-PLA_2_ activity [40,72,73,74]. *R. prowazekii* is an obligate intracellular bacterium that causes typhus fever in humans and functions to lyse red blood cells and cell membranes in a PLA-dependent manner [73,74]. Interestingly, the rickettsial ExoU RP534 orthologue only appears to be present in the typhus group of rickettsia. The remainder of the genus encodes another patatin-family PLA enzyme that has also been reported to possess an ExoU-like biochemical regulatory profile [75]. Whether rickettsial patatin-like PLAs are needed for bacterial phagocytosis into the host cell or for phagosome escape and pathogenesis has not been fully elucidated. As a second example, the complete genome sequencing of *Bacillus anthracis* (Ames strain) revealed three conserved regions that align with the glycine rich motif and serine-aspartate catalytic dyad of ExoU [40,76]. *B. anthracis* is a spore-forming Gram-positive bacterium and causative agent of anthrax [77]. The major virulence factors of *Bacillus anthracis* reside on two plasmids, pXO1 and pXO2 [77]. These plasmids encode the secreted exotoxins lethal toxin, protective antigen, and edema factor. Interestingly, the addition of PLA_2_ inhibitors (e.g., quinacrine) to mouse peritoneal macrophages reduces lethal toxin-mediated cytotoxicity [78]. Well studied PLA_2_ inhibitors, AACOCF3 (arachidonyl trifluoromethyl ketone, a selective inhibitor of cPLA_2_) and HELSS ([E]-6-[bromomethylene] tetrahydro-3-[1-naphthalenyl]-2H-pyran-2-one, an irreversible inhibitor of iPLA_2_), were no more or less potent at reducing cytotoxicity compared to quinacrine [78]. These results suggest that eukaryotic host cPLA_2_ and iPLA_2_ are not involved in lethal toxin cytotoxicity. Thus, further studies are needed to fully define the potential regulatory relationship between *the B. anthracis* ExoU PLA_2_ orthologue and lethal toxin activity.

### 2.6. ExoU Subverts the Host Innate Immune Response to Infection

*P. aeruginosa* ExoU has been associated with severe disease outcomes in animals and humans [10,35]. Infection with an ExoU expressing strain of *P. aeruginosa* is associated with poor patient outcomes and, in vitro, results in rapid cell death due to lysis. A pathogenic role for ExoU during the initial phase of the pathogen-host interaction has been described. Expression and secretion of ExoU early during the course of infection correlates with increased bacterial burden in the lungs, increased bacterial dissemination, and increased mortality [23,79,80]. Macrophages, neutrophils, epithelial, and endothelial cells are rapidly killed, allowing persistence, proliferation, and dissemination of the bacteria, ultimately contributing to sepsis [13,81]. By intoxicating and killing immune cells, ExoU dysregulates the host innate inflammatory response. Diaz and colleagues demonstrated in a *P. aeruginosa-*induced pneumonia mouse model that neutrophils are the primary immune cells recruited to the lungs during infection, and these infiltrating neutrophils are intoxicated by ExoU and killed within 90 min [82]. Macrophages are also recruited, injected with ExoU, and killed [83]. In animal infection models, ExoU associates with acute lung epithelial injury, often progressing to bacterial dissemination and septic shock [81,84]. Instillation of an ExoU-encoding strain into rabbit lungs resulted in increased epithelial permeability and leakage of pro-inflammatory mediators into the circulation, along with decreased mean arterial pressure and cardiac output indicative of septic shock. Conversely, animals instilled with a strain lacking ExoU did not exhibit signs of systemic inflammation and septic shock [81]. Furthermore, introducing the gene encoding ExoU into noncytotoxic *P. aeruginosa* strains expressing a functional T3SS, confers virulence in pneumonia animal models, often resulting in bacterial dissemination [84]. After ExoU has intoxicated and killed innate immune cells and epithelial cells, the last host defense before bacterial dissemination are endothelial cells. Pulmonary endothelial cells are an integral part of the host inflammatory response and function as a barrier between vessels and tissue restricting fluid and protein leak. ExoU has been shown to cause pulmonary endothelial barrier disruption and vasculitis in animal models of pneumonia and Acute Respiratory Distress Syndrome (ARDS) [85,86]. Interactions of T3SS effector proteins with pulmonary endothelial cells post dissemination into the pulmonary circulation are an emerging area of interest.

Recent evidence suggests the *P. aeruginosa* T3SS and ExoU also subvert a newly emerging facet of the innate immune response to infection. Amyloid-β is best known for its role in the pathology of dementias and Alzheimer’s Disease (AD). Amyloid-β is formed via sequential cleavage of the amyloid precursor protein (APP) by the β-secretase-1 and γ-secretase protease complexes [87]. Depending on the nature of the processing event, differently truncated forms of amyloid-β can be produced (e.g., amyloid-β_1–40_ or amyloid-β_1–42_). Subsequent aggregation of amyloid-β leads to the formation of cytotoxic, prion-like fibrils that self-propagate, are long-lived, and form plaques in the brains of Alzheimer’s patients [88,89,90]. Ultimately, cytotoxic amyloid-β aggregates cause neural inflammation and brain endothelial cell (EC) death [91,92,93]. More recently, amyloid-β has been incriminated as a potential contributor to lung pathology associated with pneumonia caused by the ESKAPE pathogens *P. aeruginosa*, *Staphylococcus aureus*, and *Klebsiella pneumoniae* [94]. Indeed, *P. aeruginosa*-induced pneumonia appears to trigger cytotoxic amyloid-β production in the lung as a pathological mechanism that disrupts pulmonary endothelial cell barrier function [31,33,94,95,96]. However, not all forms of amyloid-β are cytotoxic. Amyloid-β is also an antimicrobial peptide that kills a variety of pathogenic bacteria, viruses, and fungi [97,98,99]. Intriguingly, virulent *P. aeruginosa* strains expressing the T3SS and exoenzyme effectors such as ExoU inhibit production of antimicrobial amyloid-β and promote formation of cytotoxic amyloid-β as a novel mechanism to subvert the host innate immune response to infection [33]. Current studies are focused on understanding the mechanisms by which ExoU corrupts the production of antimicrobial amyloid-β.

### 2.7. ExoU Transiently Represses NLRC4 Inflammasome Activation

The innate immune response is a critical first line of defense to invading pathogens or cellular stress. During infection, pathogen- and damage-associated molecular patterns (PAMPs and DAMPs, respectively) are sensed by innate pattern recognition receptors (PRRs), which then assemble with accessory proteins to form an inflammasome. Inflammasomes are multi-protein complexes of PRRs that sense PAMPs and DAMPs produced during infection or other conditions of cellular stress [100]. The nucleotide-binding domain containing leucine rich repeats-like receptor 4 (NLRC4) inflammasome has been shown to recognize *P. aeruginosa* through interactions between NLR family apoptosis inhibitor proteins (NAIP) receptors and bacterial ligands (e.g., NAIP1-T3SS needle, NAIP2-T3SS rod, and NAIP5/6-flagellin) [101,102,103,104]. In turn, the activated NLRC4 inflammasome complex assembles to subsequently convert pro-caspase-1 into its activated form. Caspase-1 activation results in maturation of IL-1β, IL-18, and the gasderminD executioner of pyroptotic cell death. Activation of pyroptosis and cell death facilitates release of IL-1β, IL-18, and pro-inflammatory DAMPs the extracellular milieu, which then triggers a feed-forward inflammatory response.

A dysregulated host inflammatory response is a hallmark of poor patient outcome progressing to ARDS and sepsis [105,106]. Sutterwala and colleagues first demonstrated recognition of *P. aeruginosa* by the NLRC4 inflammasome during infection [107]. Importantly, ExoU transiently paralyzes caspase-1 driven NLRC4 inflammasome activation within the first 2 h of *P. aeruginosa* infection [85,107]. However, the underlying mechanism by which ExoU transiently paralyzes caspase-1 driven NLRC4 inflammasome activation has not been defined. Previous studies have demonstrated a link between *P. aeruginosa* T3SS, NLRC4 inflammasome, and autophagy. During *P. aeruginosa* infection, mitochondria become damaged activating the NLRC4 inflammasome. Mitochondria-specific autophagy (mitophagy) is induced in response to mitochondrial damage, which, in turn, down-regulates NLRC4 inflammasome activation [108]. Future studies are required to fully elucidate a potentially novel pathway through which ExoU dysregulates the host inflammatory response during *P. aeruginosa* infection.

## 3. Perspectives

*P. aeruginosa* strains expressing the ExoU PLA_2_ exoenzyme effector are known to associate with the most adverse patient outcomes, likely owing to the ability of ExoU to subvert host innate immune responses and cause cellular damage. Virulence factors, such as the T3SS and ExoU, represent the next frontier of precision medicine therapeutic targets to combat the imminent threat of antibiotic resistant infectious agents such the ESKAPE pathogens. ExoU is an especially attractive potential target, considering its contributions to *P. aeruginosa* virulence and its requirement for a eukaryotic-specific protein, ubiquitin, to fully hydrolyze membrane substrates. As the substrate specificity of ExoU is relatively broad, the interaction between ExoU and SpcU, as well as the requirement for interaction with ubiquitin for enzyme activation, are postulated to protect *P. aeruginosa* from the membrane destructive effects of ExoU prior to its direct injection into host cells by the T3SS. Thus, interventions that activate ExoU in the bacteria or inactivate ExoU in the host could mitigate *P. aeruginosa* infection. In addition, ExoU orthologs and related enzymes that also require similar activation steps are encoded by a variety of Gram-negative bacteria that are pathogenic for humans. Understanding the structural transitions between inactive and active states of these proteins may provide a target for rationally designed ligands that can prevent this conversion to a cell and tissue destructive virulence factor. The availability of crystal structures and recent use of biophysical and computational modeling techniques [54] open new horizons for understanding allosteric changes of dynamic enzymes in response to both cofactors and substrates.

## Data Availability

Not applicable.
